# Crocin attenuates cigarette smoke-induced lung injury and cardiac dysfunction by anti-oxidative effects: the role of Nrf2 antioxidant system in preventing oxidative stress

**DOI:** 10.1186/s12931-018-0766-3

**Published:** 2018-04-10

**Authors:** Mahin Dianat, Maryam Radan, Mohammad Badavi, Seyyed Ali Mard, Vahid Bayati, Masoumeh Ahmadizadeh

**Affiliations:** 1 Department of Physiology, Physiology Research Center, Faculty of Medicine, Ahvaz Jundishapur University of Medical Sciences, Ahvaz, Iran; 2 Cellular and Molecular Research Center, Faculty of Medicine, Ahvaz Jundishapur University of Medical Sciences, Ahvaz, Iran; 3 Physiology Research Center, School of Health, Ahvaz Jundishapur University of Medical Sciences, Ahvaz, IR Iran

**Keywords:** COPD, Cigarette smoke, Crocin, Oxidative stress, Hemodynamic parameters, Inflammatory parameters, Nrf2, GCLc, GSH

## Abstract

**Background:**

Chronic obstructive pulmonary disease (COPD) has been emerging as a great health problem in world. Cigarette smoke is known to cause oxidative stress and deplete glutathione (GSH) levels. Nuclear erythroid-related factor 2 (Nrf2) is involved in transcriptional regulation of glutamate-cysteine ligase catalytic subunit (GCLc). Antioxidant compounds may be of therapeutic value in monitoring disease progression. Crocin demonstrates antioxidant and anti-inflammatory functions. The aim of this study was to investigate the protective role of crocin against CSE-mediated oxidative stress, inflammatory process, Nrf2 modifications and impairment of cardiac function in rats with COPD.

**Methods:**

Eighty rats were divided into four groups: Control, Cigarette smoke exposure (CSE), Crocin, Crocin+CS. Each group was divided into the two parts: 1) to evaluate lung inflammatory and oxidative process, 2) to evaluate the effect of Cigarette smoke induced-lung injuries on cardiac electrocardiogram (such as heart rate and QRS complex) and hemodynamic parameters (such as perfusion pressure and left ventricular developed pressure).

**Results:**

CSE rats showed a significant increase in cotinine concentration (17.24 ng/ml), and inflammatory parameters and a decrease in PO_2_ (75.87 mmHg) and expression of PKC (0.86 fold), PI3K (0.79 fold), MAPK (0.87 fold), Nrf2 (0.8 fold) and GCLc (0.75 fold) genes, antioxidant activity, and finally cardiac abnormalities in electrocardiogram and hemodynamic parameters. Co-treatment whit crocin could restore all these values to normal levels.

**Conclusions:**

CS induced-COPD in rat model provides evidence that chronic CS exposure leads to lung injury and mediated cardiac dysfunction. Crocin co-treatment by modulating of Nrf2 pathway protected lung injury caused by COPD and its related cardiac dysfunction. In this study, we showed the importance of Nrf2 activators as a therapeutic target for the development of novel therapy for lung oxidative injuries.

## Background

Chronic obstructive pulmonary disease (COPD) as a global health problem is a major cause of morbidity and mortality that is set to become the third leading cause of death worldwide by 2020 [1].

There is overwhelming evidence that oxidative stress plays an essential role in the pathogenesis of COPD [2]. This oxidative damage to cellular organelles plays a central role in mediating a wide array of downstream processes that contribute to the development and progression of COPD. Oxidative stress also stimulates epithelial cells and alveolar macrophages to generate additional inflammatory cells into the lung which resulting in severe pulmonary injury [3, 4]. Increased oxidative stress in the respiratory compartment of COPD patients originates from the increased burden of oxidants from environmental exposures such as air pollutants, cigarette smoke (CS), and increased amounts of reactive oxygen species (ROS) released from inflammatory cells involved in the destructive inflammatory process in the lungs of COPD subjects [5]. Exposure of macrophages and lymphocytes to oxidative stress results in release various inflammatory substances including cytokines and chemokines, which could destroy collagen and elastin, stimulate mucosal secretions in lung tissues, and even lead to more destructive processes in lungs [6].

Cigarette smoking is the major risk factor for the development of COPD. CS contains more than 5000 different chemicals and generates more than 10^15^ oxidants per puff, directly or indirectly, through various processes such as the Haber-Weiss reaction [7]. Reactive oxygen species, in turn, can induce lipid peroxidation and yield products such as malondialdehyde (MDA), which have the ability to stimulate pulmonary inflammation [8].

Under normal physiological conditions oxidant challenge is normally neutralized by the antioxidants in the epithelial lining fluid. Oxidative stress occurs if antioxidant levels in the epithelial lining fluid are inadequate to neutralize the inhaled oxidants/free radicals [9]. Among various antioxidants the reduced glutathione (GSH), as the most abundant cellular thiol antioxidant, plays an essential role in the maintenance of intracellular redox balance in epithelial lining fluid and is involved in the detoxification reaction through enzyme-catalyzed reactions or by direct conjugation [10]. This critical antioxidant has been reported to be depleted in pulmonary disorders, such as cystic fibrosis, acute respiratory distress syndrome, and COPD, suggesting a role for oxidative stress in the pathogenesis of these chronic inflammatory lung diseases [11, 12].

The synthesis of GSH depends on the rate of synthesis of the GCL catalytic (GCLC) subunit through increased transcription and mRNA stability [13].

Nuclear erythroid-related factor 2 (Nrf2), a member of the cap-N-collar family, is the principal transcription factor that regulates antioxidant response element-mediated expression of antioxidant enzymes. Under basal conditions, Nrf2 is sequestered in the cytoplasm by actin-binding protein (Keap1); on exposure of lungs to oxidative stress, Nrf2 dissociates from its repressor protein (Keap1), translocates into the nucleus, binds to antioxidant response elements, and transactivates antioxidant genes [14]. Nrf2 phosphorylation is mediated by protein kinase C (PKC), phosphoinositide-3-kinase (PI3K) and mitogen-activated protein kinases (MAPK). The modifications of each of the kinases in Nrf2 activation depends on the stress stimuli [15]. Nrf2 play a critical role in induction of multiple cytoprotective genes. One of the genes regulated by Nrf2 is heme oxygenase-1 (HO-1). The induction of HO-1 was able to exert protective role against oxidative stress [16]. Several experiences demonstrated a reduced expression of HO-1 related to an altered expression of Nrf2 in cigarette smoke related lung disease such as COPD [17–19]. This process has pathophysiological roles since it decreases endogenous antioxidant capacity [16]. Among the spectrum of antioxidant genes controlled by Nrf2 pathway, the gene encoding GCLc is of particular interest, especially in CS-induced oxidative stress [20]. Therefore, agents that modulate Nrf2 as Nrf2 activators would be expected to have significant beneficial health effects in CS-mediated oxidative stress by upregulation of antioxidant enzymes.

In addition to pathology in the lungs, COPD is now believed to have systemic features. An increase in the risk of cardiovascular disease is one such systemic feature. There is considerable evidence of an association between COPD and cardiac disease [21]. The range of cardiovascular disease includes coronary artery disease (CAD), right ventricular dysfunction and arrhythmias. Cardiac disorders associated with COPD increase morbidity and worsen survival. Patients with COPD also carry an increased risk of mortality due to myocardial infarction, arrhythmia or congestive heart failure compared with those who do not. Since the cardiac dysfunction and abnormalities obviously contribute to the overall morbidity associated with COPD [22], therefore, an understanding of their role and potential for treatment is necessary.

Saffron (*Crocus sativus* L.) has four major pharmacologically active constituents, namely crocetin, picrocrocin, safranal and crocin. Crocin is a water soluble carotenoid and the most important active constituent of saffron. In pharmacological studies, crocin has demonstrated anti-inflammatory, anticonvulsant and anti tumour activities. Radical scavenger effects as well as learning and memory improving properties [23], and it is reported to promote the diffusivity of oxygen in different tissues [24]. Crocin is also chemopreventive and has shown protective effects on genotoxins-induced oxidative stress in Swiss albino mice [25]. Current studies have shown that crocin exhibits significant radical scavenging activity and thus antioxidant activity. Moreover, the cardio protective effects of crocin have been documented in some studies in relation to modulating endogenous antioxidant enzymatic activities [26, 27].

In light of the findings described above, we hypothesize that crocin induces GSH Synthesis via Nrf2 dependent mechanisms and attenuates cigarette smoke-induced COPD mediated oxidative stress in lung tissue that leads to cardiac dysfunctions. Therefore, we investigated the protective role of crocin against cigarette smoke exposure (CSE)-mediated oxidative stress and the inflammatory process in COPD, modifications of Nrf2 and its upstream regulator genes (PKC, PI3K AND MAPK) and also GCLc and GSH as downstream enzymes controlled by Nrf2 and impairment of cardiac hemodynamics and remodeling in rats with COPD.

## Methods

### Materials

Crocin was purchased from Sigma-Aldrich Co. (USA). Ketamine HCl (10%) and Xylazine (2%) were obtained from Alfasan Co. (Netherlands). Krebs salts were purchased from Merck Co. (Germany). Antioxidant assay kits were purchased from ZELLBIO (Germany). Cytokines Elisa kits were purchased from DIACLONE (France).

### Animals

Eighty male Sprague-Dawley rats (180–200 g) were purchased from the Ahvaz Jundishapur University of Medical Sciences Animal Lab. For prevention of pneumonia, rats were housed under pathogen-free conditions with ad libitum access to food and water and subjected to a light–dark cycle of 12 h.

### Experiments

This experiment was divided into the two parts. The first part of this investigation dealt with approval processes for establishment of cigarette smoke –induced-lung injuries model in rats and evaluation of inflammatory and oxidative processes induced in this group. The second part dealt with the effect of lung injuries model on cardiac electrocardiogram and hemodynamic parameters. In both groups, we evaluated the protective effect of crocin in prevention of lung and cardiac dysfunction.

### Part I

#### Establishment of CS-induced lung injuries model

The animals were randomly divided into four groups, 10 rats each:

1. Fresh air (Control group)
2. Cigarette smoke exposure (CS) generated by Winston Red Cigarettes, R.J. Reynolds Tobacco Company, USA (nicotine: 1 mg),
3. Crocin [28] (50 mg/kg, intraperitoneally, three times per week, once a day for 2 months).
  [Concentration-effect study (12.5, 25 and 50 mg/kg, IP) was performed with crocin to determine the effective dose. In lung tissue, crocin 12.5 mg/kg had no effect on MDA level, but crocin 25 (*P* < 0.05) and 50 mg/kg significantly (*P* < 0.001) inhibited lipid peroxidation. Crocin at 50 mg/kg was found to effectively decrease MDA as oxidative stress index].
4. Crocin co-treatment CS.
  In the CS- induced lung injuries model group, the rats were completely exposed to smoke for 2 months, daily, four cigarettes per day (twice a day in the morning and evening). During this period, the behavior, morphology, and fatality rate were considered. In addition, lung markings and lung tissue morphology were both evaluated by histology and chest x-ray film (Varian medical system, 985H, USA). The characteristics such as obvious thickenings and structural changes were considered diagnostic for COPD.

In the CS- induced lung injuries model group, the rats were completely exposed to smoke for 2 months, daily, four cigarettes per day (twice a day in the morning and evening). During this period, the behavior, morphology, and fatality rate were considered. In addition, lung markings and lung tissue morphology were both evaluated by histology and chest x-ray film (Varian medical system, 985H, USA). The characteristics such as obvious thickenings and structural changes were considered diagnostic for COPD.

#### Cigarette smoke exposure system

**Fig. 1:**
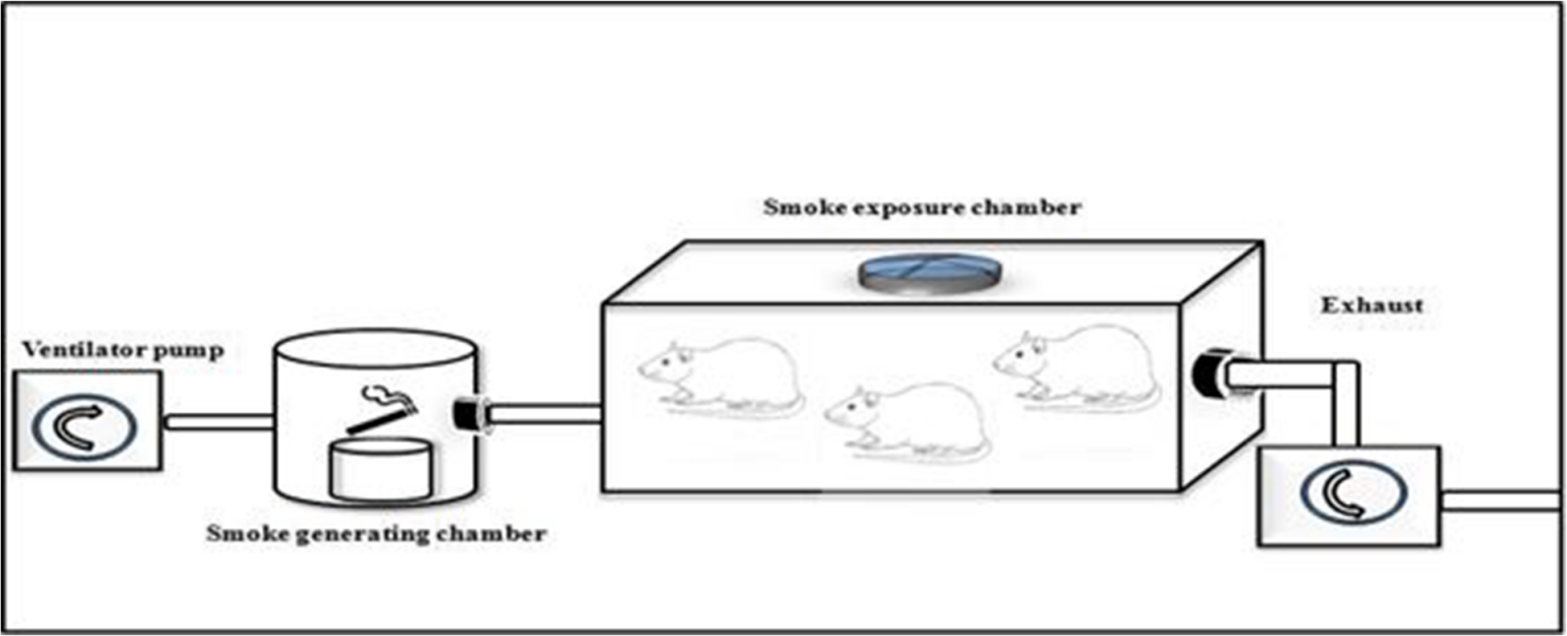
The smoke-exposure system

The system used to expose rats to sidestream cigarette smoke consisted of a peristaltic pump, a smoke-generating chamber, and a whole-body CSE chamber that were serially connected via silicone tubes (Fig. 1). The ventilator pump (UGO BASILE, model: 7025) was set to supply 150 mL of air every 10 s. The smoke-generating chamber consisted of an acrylic cylinder (height, 27 cm; diameter, 16 cm) corresponding to 5430 cm3 total volume into which one cigarette at a time was constantly kept lit. Smoke then was delivered to an inhalation chamber (length, 40 cm; width, 20 cm; height, 25 cm) of 20,000 cm^3^ total volume and exhausted through a hole. Also, the system contained a fan to circulate the air into the chamber. The carbon monoxide in air was collected from the chamber interior at 15-min intervals [28].The total particulate matter (TPM) concentrations in this study was measured daily and indicated an average of 400 mg total particulate matter per m^3^.

#### Determination of serum cotinine concentration

Cotinine is a metabolite of nicotine and is the primary biomarker for the determination of cigarette smoke exposure. Blood samples were collected under anesthesia. Collection of blood samples for cotinine measurement in rats was performed 1 h after the last session in CS and control group [29]. Serum was separated by centrifugation at 3000×*g* for 20 min and then stored at − 80 °C until later analyzed. The concentration of cotinine was determined by using a commercially available ELISA kit (Cotinine ELISA kit, ZellBio, Germany) according to the manufacturer’s instructions. The lower limit of detection was 0.02 ng/ml.

#### Arterial blood gas analysis and bronchoalveolar lavage fluid assay

The rats were anesthetized with Xylazine 10 mg/kg and Ketamine 50 mg/kg. The abdominal cavity was open and blood was collected from abdominal aorta using heparinised syringes. All blood samples for arterial blood gas analysis were immediately analysed by blood gas analyzer. Then, thoracotomy was done. The lungs of rats were instilled with 1 ml of phosphate-buffered saline (PBS) and the fluid was gently aspirated back. This procedure was repeated for three times. The BALF was centrifuged at 1000 g for 10 min at 4 °C, and the supernatant was stored immediately at − 80 °C until analysis [30]. The cell pellet was resuspended in PBS, and cell suspension was made into the smear on a glass slide. The cell smear was air-dried and stained with wright-giemsa solution. The total and differential leukocyte counts were determined under 400× magnification.

#### Protein determine in BALF

The protein concentration in bronchoalveolar lavage fluid was measured by Bradford’s method and bovine serum albumin (BSA) was used as the standard according to the Lowry method [31].

#### Cytokines analysis in BALF

The levels of tumor necrosis factor (TNF)-α and interleukin (IL)-6 in BALF supernatant were measured by ELISA kit (Zell Bio, Germany), according to manufactures’ instruction on the basis of the Biotin double antibody sandwich technology.

#### Lung water content

The left lower lung was cut off and weighed prior to and following drying in an oven at 80 °C for 48 h. The water content was calculated as the (wet weight/ dry weight) and used to represent the pulmonary edema [32].

#### Lung histopathological examination

After 2 months of treatment, the lung tissue was removed from the rats of different groups, fixed, paraffin embedded, sectioned at 4 μm and stained with hematoxylin and eosine (H&E) for microscopic examination of morphological changes [33]. Also, the mean linear intercepts (MLI) evaluated to determine airspace enlargement within the lung parenchyma in all groups. Twenty randomly selected fields in each section at 200× magnification were utilized to calculation of MLI using a 21 line counting grid.

#### Preparation of lung tissue for measuring antioxidant enzymes

The rat lungs were removed and manually homogenized in cold PBS. After centrifugation, the supernatant was removed and serum enzymes were measured by the standard commercial kits.

##### Measurement of malondialdehyde

Malondialdehyde (MDA) is the end product of the major chain reactions leading to oxidation of fatty acids, and measurement of MDA content is the most widely used method for assessing lipid peroxidation. The amount of lipid peroxidation was assessed by thiobarbituric acid reactive substances (TBARS) in the lung. MDA activity was determined colorimetrically at 532 nm. All parameters were evaluated using diagnostic Zell Bio kits and according to the manufacturer’s instructions (Zell Bio, Germany).

##### Measurement of glutathione amount

The GSH amount unit was considered as the amount of the sample that will catalyze decomposition of 1 μmole of GSH to GSSG in one minute. GSH was determined calorimetrically at 412 nm according to the manufacturer’s instructions (Zell Bio, Germany).

##### Measurement of super oxide dismutase activity

The unit of SOD activity (as antioxidant enzyme) was expressed as the amount of the sample that will catalyze 1 μmole of superoxide radicals to hydrogen peroxide and oxygen in one minute. SOD activity was determined colorimetrically at 420 nm according to the manufacturer’s instructions (Zell Bio, Germany).

##### Measurement of glutathione peroxidase activity

In this method, the GPX activity unit was expressed as the amount of the sample that will catalyze 1 μmole GSH to GSSG in one minute. GPX activity was determined calorimetrically at 412 nm according to the manufacturer’s instructions (Zell Bio, Germany).

##### Measurement of catalase activity

The CAT activity unit was considered as the amount of the sample that will catalyze 1 μmole of hydrogen peroxide to water and oxygen in one minute. Catalase activity was determined colorimetrically at 405 nm according to the manufacturer’s instructions (Zell Bio, Germany).

#### RNA extraction and cDNA synthesis

The total RNA was extracted from the frozen lung tissue samples using TriPure reagent isolation (Roche, Diagnostics). The purity and concentration of the extracted RNA were determined spectrophotometrically at 260 and 280 nm wavelengths (Eppendorf, BioPhotometer Plus, Germany). Then, the cDNA was synthesized from one microgram of the total RNA using a cDNA synthesis kit (Qiagen, USA) according to the manufacturer’s instruction.

##### Quantitative real-time RT-PCR

We used real-time PCR (RT-PCR) to determine the transcriptional induction of antioxidant genes including Nrf2 (principal transcription factor that regulates antioxidant response element-mediated expression of antioxidant enzymes), GCLc and HO-1 (as a downstream gene controlled by Nrf2) and upstream Nrf2 regulator genes (PKC, PI3K and MAPK) and also cytokines gene such as IL-6 and TNF-α in the lungs of all rat groups. The total RNA was extracted from the frozen tissue samples using TriPure reagent isolation (Roche, Diagnostics). The purity and concentration of the extracted RNA were determined spectrophotometrically at 260 and 280 nm wavelength (Eppendorf, BioPhotometer Plus, Germany). The cDNA was synthesized from one microgram of the total RNA using a cDNA synthesis kit (Bioneer, Daejeon, South Korea) according to the manufacturer’s instruction. The specific primers (Bioneer, Daejeon, South Korea) were used and the lengths for amplified products are shown in Table 1. The mRNA levels of the target (Nrf2, GCLc, HO-1, PKC, PI3K, MAPK, IL-6 and TNF-α) along with glyceraldehyde-3-phosphate dehydrogenase (GAPDH), were measured by quantitative real-time PCR (qPCR) using step-one systems (Applied Biosystems, USA). All PCR amplifications were performed in duplicate reactions and in final volume of 20 μL containing 2 *μ*L cDNA, 50 nm of specific primers, and 10 *μ*L of Master Mix SYBR Green (2× qPCR Master Mix with SYBR Green I and Rox; Primer design, England) using the following protocol: incubation at 95 °C for 10 min to activate DNA Taq polymerase, 40 two-step cycles with 15 s at 95 °C for denaturation, and annealing-extension at 60 °C for 1 min. In addition, the no-template negative control (H_2_O) was routinely run in every PCR. The melting curve was examined at the end of amplification process to ensure the specificity of PCR products. Expression levels of all Nrf2, HO-1, GCLc, PKC, PI3K, MAPK, IL-6 and TNF-α gene were normalized against GAPDH expression. To determine the relative quantification of gene expression, comparative cycle of threshold (Ct) method with arithmetic formulae (2 − ΔΔCt) was used [34]. Expression in control animals was normalized to 1. GAPDH was used for normalization, and all PCRs were assayed in duplicate.

**Table 1:** Sequences of the Different Primers

mRNA	Sense Primers	Antisense Primers
Nrf2	CTCTCTGGAGACGGCCATGACT	CTGGGCTGGGGACAGTGGTAGT
GCLc	GTGGACACCCGATGCAGTAT	TCATCCACCTGGCAACAGTC
HO-1	CGTGCAGAGAATTCTGAGTTC	AGACGCTTTACGTAGTGCTG
TNF-α	ACTGAA CTT CGG GGT GAT TG	GCT TGG TGG TTT GCT ACG AC
IL-6	TGATGG ATG CTT CCA AAC TG	GAGCAT TGG AAG TTG GGG TA
PKC	TGGACCCCACGACAACTTTC	ACTTCGTCCCTGCCCTGAC
MAPK	GGAGCAGTATTATGACCCAAGTGA	TCGTCCACTCCATGTCAAACT
PI3K	AACACAGAAGACCAATACTC	TTCGCCATCTACCACTAC
GAPDH	GTATTG GGC GCC TGG TCA CC	CGCTCCTGGAAGATGGTGATGG

### Part ii

#### Electrocardiogram recording method

After the procedures, the animals were anesthetized. Rectal temperature was continuously monitored and maintained within 37–38 °C using a heat pad and heat lamp. Lead II electrocardiogram was recorded (PowerLab, ADInstruments, Australia) using three 26-gauge needles surface electrodes. The electrodes were connected to a Bioamp amplifier (ADInstruments, Australia) and were digitalized through an A/D converter PowerLab 8sp (ADInstruments, Australia). Digital recordings were analyzed with Chart software (ADInstruments, Australia). Events were registered to 4 K/s and filtered to 60 Hz20. The ECG was calibrated for 25 mm/s with a sensitivity of 10 mm = 10 mV. The following parameters were assessed, namely, the heart rate, the RR interval and the QRS complex duration and voltage. The QTc (corrected QT interval) was calculated using Bazett’s formula (QT interval/square root of the RR interval) [35]. The QT interval and the ST segment were evaluated in seconds.

#### Langendorff-perfused heart preparation for cardiac function

The rats were anesthetized (ketamine, xylazine and heparin). The trachea was cannulated and the rats were ventilated by room air using a rodent ventilator (UGO BASILE, model: 7025). A mid sternal thoracotomy was performed and a steel cannula inserted through an aortotomy into the aorta and secured by a suture. The hearts were immediately perfused using Krebs-Henseleit bicarbonate buffer at a constant pressure of 60–70 mmHg and a temperature of 37 °C. Buffer was bubbled using 95% O2–5% CO2 to attain a pH of 7.4. The heart was quickly excised from the chest and transferred to a Langendorff apparatus while continuously perfused. A water-filled latex balloon attached to a pressure transducer by a stainless steel needle was inserted through the left atrium into the left ventricle for measuring left ventricular pressure (LVP). The heart was submerged in a jacketed, temperature-controlled glass chamber and allowed to equilibrate for 25–30 min. The balloon volume was set to maintain a left ventricular end diastolic pressure (LVEDP) of 5 mmHg. The signal from the pressure transducer was analyzed using a Power Lab system (ADInstruments, Australia). Heart rate (HR), left ventricular end systolic pressure (LVESP), left ventricular end diastolic pressure (LVEDP), perfusion pressure, left ventricular developed pressure (LVDP: LVSP-LVEDP), maximum rate of rise (dp/dt_max_) of LVP and rate-pressure product (calculated as HR × LVSP) were measured. Meanwhile, HR and perfusion pressure were continuously monitored [36].

#### Assessment of right ventricular hypertrophy

The hearts were removed and dissected to isolate the free wall of the right ventricle from the left ventricle and septum. The ratio RV/LV + S (Fulton’s index) was used as an index of cardiac hypertrophy [37].

#### Statistical analysis

All data were expressed as Mean ± SEM. Differences between experimental groups were analyzed by one-way ANOVA and followed by post hoc Tukey’s test.

## Results

### Confirmation of CS-induced lung injuries model in rat

**Fig. 2:**
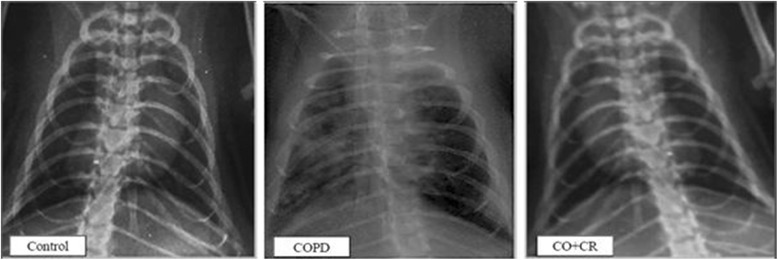
Chest x-ray film observation of lung tissue in Control, COPD and COPD plus crocin (CO + CR) rats

With the purpose of establishing the model of CS- induced COPD, the rats were exposed to cigarette smoke for 8-weeks. As a result, cough was first detected in 10% of rats in the first 2 weeks. Then the rate of cough reached to 30% of rats in the third week, and laryngeal stridor began to appear in 20% of rats. After 4 week of treatment, all rats exhibited the symptoms of cough, and 60% of the rats exhibited the symptoms of laryngeal stridor with considerable nasal secretion. Additionally, hypokinesia, flagging spirit and reduced weight were also observed in these rats. The evaluation of chest x-ray showed 90% of rats had significantly diffuse patchy alveolar infiltration, hyperinflation and disordered lung markings, suggesting that the model of CS-induced lung injuries was successfully established in cigarette smoke exposure group (Fig. 2). Pneumonia was not seen in any of rat groups.

### Serum cotinine level

**Fig. 3:**
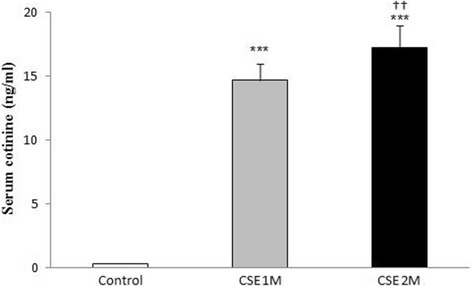
Mean serum cotinine concentration (*n* = 8) of rats at 1 h after daily smoke exposure during the CSE period (1 and 2 Month). CSE, cigarette smoke exposure; ^***^P < 0.001 vs. control. ^†††^*P* < 0.001 difference between CSE 1 M and CSE 2 M. Data was analyzed using SPSS by t-test

Serum cotinine levels at 1 h after last cigarette smoke exposure in the first and the second months were 14.7 ± 1.25 and 17.24 ± 1.77 ng/ml respectively, which were significantly (*P* < 0.001) higher in CS group compared to the control group (Fig. 3), and this suggests that the CS exposure setup induced a reliable and reproducible increase in serum cotinine.

### Results of blood gas analysis

ABG provides the best criteria for diagnosis and severity of COPD. After 2 months and in the last session, arterial blood gas concentration was measured in all rat groups. PO_2_, PCO_2_ and pH in CS rats had statistically different concentrations compared with control rats (Table 2). These variables were used to confirm the efficacy of the exposure of smoking animals to cigarette smoke. However, CS rats co-treated with crocin had a significantly higher partial pressure of oxygen; lower partial pressure of CO_2_ and pH than did rats exposed to smoking (CS group).

### Protein estimation in BALF

**Fig. 4:**
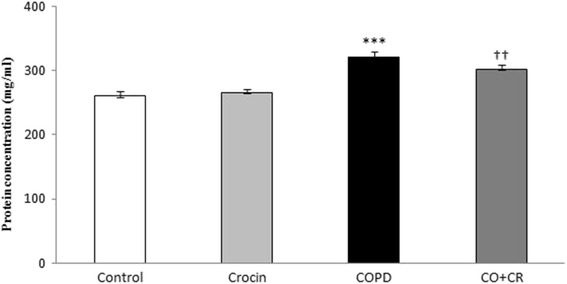
Total protein measurment in BALF. ***P < 0.001 compare to control, ^††^*P <* 0.01 compare to COPD group.Values are expressed as mean ± SEM (*n* = 6); all the groups were statistically compared by ANOVA followed by Tukey’s multiple comparison

The CS group showed a significant elevation of total protein concentrations in the BALF (Fig. 4) compared to the control group (*p* < 0.001). While, crocin co-treatment significantly reduced the cigarette smoke-induced enhanced of total protein levels in the BALF (*p* < 0.01).

### Inflammation cell count in the BALF

**Fig. 5:**
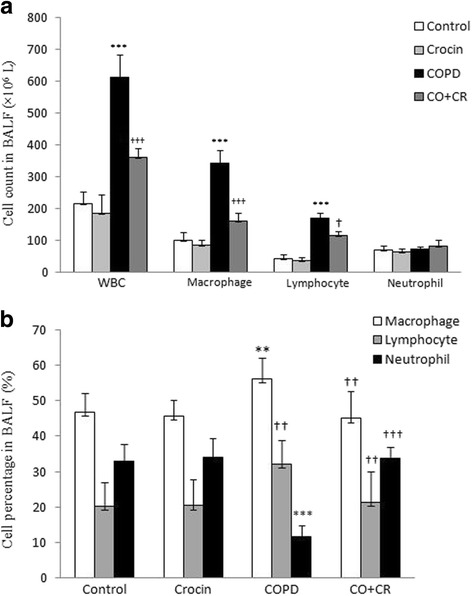
Total cell count and classification in bronchoalveolar lavage fluid. **P < 0.01 and ***P < 0.001, vs. control, ^†^*P* < 0.05, ^††^*P* < 0.01 and ^†††^*P* < 0.001 vs. COPD. Data are expressed as the mean + SEM, *n* = 8. All the groups were statistically compared by ANOVA followed by Tukey’s multiple comparison. Data are expressed as the mean + SEM, *n* = 8

As shown in Fig. 5, the total cell count and the absolute (A) and percentage (B) of macrophages and lymphocytes cell of CS rats were significantly higher than those of the control group (*P* < 0.001 and *P* < 0.01). Compared with CS group, the BALF total cells, macrophages and lymphocyte cell in crocin co-treatment CS group decreased remarkably and the differences were of statistical significance (*P* < 0.01).

### BALF contents of IL-6 and TNF-a

**Fig. 6:**
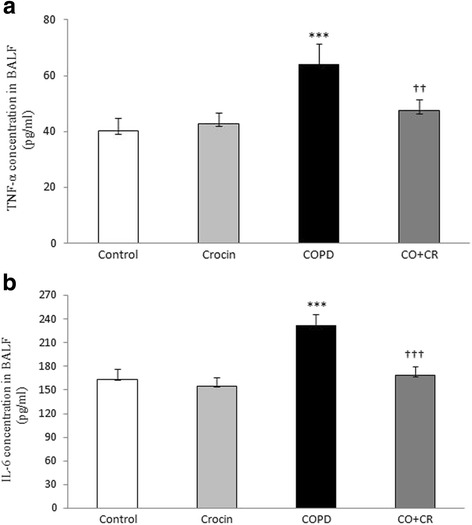
BALF contents of interleukine-6 (IL-6) and tumor necrosis factor-a (TNF-a) in Control, Crocin, COPD and COPD plus crocin (CO + CR) rats. ***P < 0.001 vs. the Control group; ^†††^*P* < 0.001 vs. the COPD group. All the groups were statistically compared by ANOVA followed by Tukey’s multiple comparison. Data are expressed as the mean + SEM, *n* = 8

To evaluate the inflammatory changes in all groups, the BALF contents of IL-6 and TNF-α were analyzed. According to the results, there was a significant increase in IL-6 and TNF-α induced after CS treatment (*P* < 0.001). However, the levels of IL-6 and TNF-α remained at normal values by co-administration of crocin in CS rats (Fig. 6).

### Lung water content

**Fig. 7:**
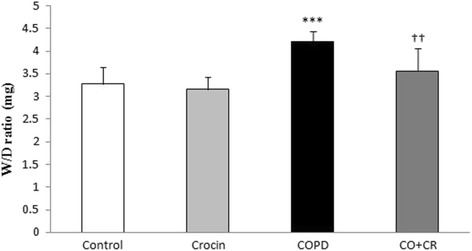
Lung wet-to-dry ratio in Control, Crocin, COPD and COPD plus crocin (CO + CR) rats. ***P < 0.001 vs. the Control group; ^††^*P* < 0.001 vs. the COPD group. All the groups were statistically compared by ANOVA followed by Tukey’s multiple comparison. Data are expressed as the mean + SEM, *n* = 8

To determine the degree of pulmonary edema, we detected the wet/dry ratio which reflects the water content of the lung. After 2 months of cigarette smoke exposure, water content in the lung in model group (CS rats) was higher than that in the control group (*P* < 0.001), while crocin co-treatment CS group showed a significantly lower lung water content than did CS group (Fig. 7).

### Lung histological changes

**Fig. 8:**
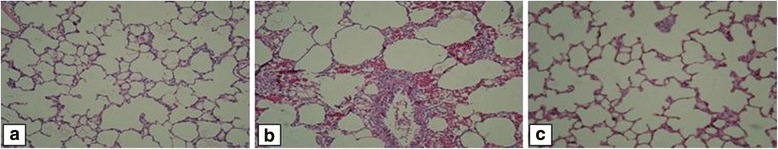
Representative Hematoxylin and Eosin (H&E) staining of lung tissue in Control, COPD and COPD plus crocin (CO + CR) rats. Scale bar = 200 μm

**Fig. 9:**
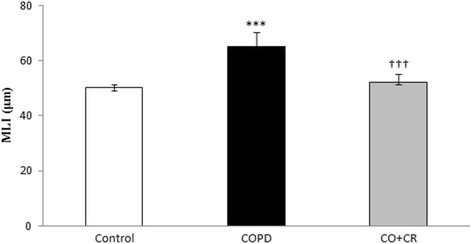
Mean linear intercept (MLI) is reported as a measurement of alveolar airspace enlargement in Control, COPD and COPD plus crocin (CO + CR) rats. ***P < 0.001 vs. the control group and ^†††^*P* < 0.001 vs. the COPD group. All the groups were statistically compared by ANOVA followed by Tukey’s multiple comparison. Data are expressed as the mean + SEM, *n* = 8

HE-staining was performed to further evaluate the pathological changes in the lung tissue, on each group. According to Fig. 8, the lung tissue was significantly damaged in cigarette smoke exposure rats, in which there were severe damage manifested, degeneration of bronchial mucosal epithelium cells, increased airspace and inflammation in bronchial wall. However, airspace enlargement was measured by mean linear intercept (MLI). There is significant increases in MLI after 2 month cigarette smoke exposure (Fig. 9) compared to control group (*p* < 0.001). The mean linear intercept was decreased in crocin-co treatment group as compared to CS rats.

### Lipid peroxidation and antioxidant enzymes activity in the lung tissue

**Fig. 10:**
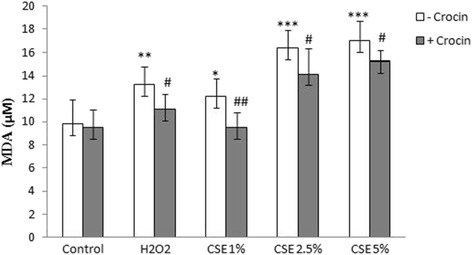
MDA level in lung tissue of Control, Crocin, COPD and COPD plus crocin (CO + CR) rats. ***P < 0.001 vs. the control group and ^††^*P* < 0.001 vs. the COPD group. All the groups were statistically compared by ANOVA followed by Tukey’s multiple comparison. Data are expressed as mean ± SEM, *n* = 8

Following 2 months of cigarette smoke exposure, the lung tissue level of MDA significantly increased as compared to the control rats (*P* < 0.001). Free radical-induced lipid peroxidation significantly decreased as indicated by a reduction in the MDA levels of lung tissue by crocin co-treatment when compared to CS group (*P* < 0.01, Fig. 10).

**Fig. 11:**
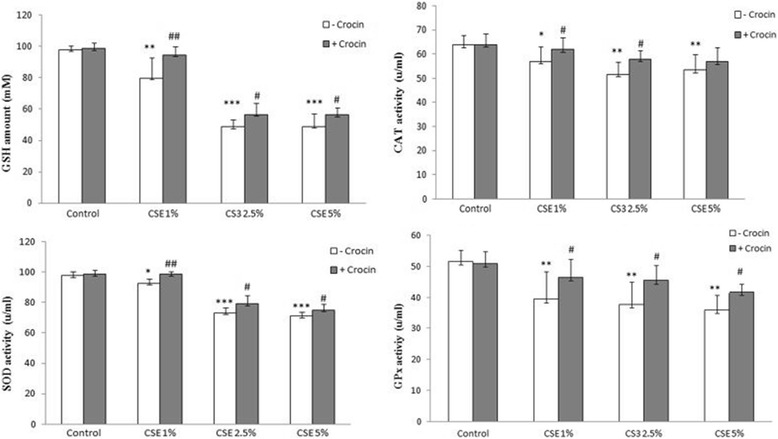
GSH amount and CAT, SOD and Gpx activities in lung tissue of the Control, Crocin, COPD and COPD plus crocin (CO + CR) rats. **P < 0.01, ***P < 0.05 vs. the Control group and ^†^*P* < 0.05, ^††^*P* < 0.01 ^†††^*P* < 0.001 vs. the COPD group. All the groups were statistically compared by ANOVA followed by Tukey’s multiple comparison. Data are expressed as mean ± SEM, *n* = 8

The aim of this study was to determine the GSH amount (downstream antioxidant enzyme for ROS-Nrf2-GCLc-GSH pathway), but activities of the other antioxidant enzymes were measured too. As shown in Fig. 11, the amount of GSH and activity of SOD, CAT and Gpx in tissue of the lung in CS group were significantly lower than that in control animals. These levels significantly increased by co- administration of crocin in CS group.

### RT-PCR for antioxidant and cytokines gene expression

As an oxidative stress sensing genetic transcription factor, Nrf2 appears to be a master regulator of cellular responses to oxidative damage and other stressful conditions. We evaluated the levels of Nrf2 gene expression and its downstream antioxidant enzyme GCLc for assaying the changes in these genes in response to cigarette smoke exposure and crocin treatment, in all groups.

**Fig. 12:**
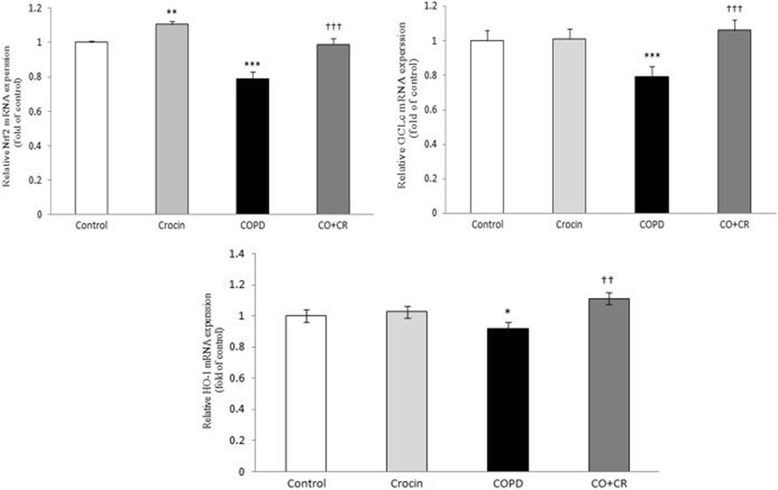
Nrf2, HO-1 and GCLc mRNA expression in lung tissue of Control, Crocin, COPD and COPD plus crocin (CO + CR) rats. *P < 0.05,***P < 0.01 vs. Control rats. ^††^*P* < 0.01, ^†††^*P* < 0.05 vs. the COPD group. All the groups were statistically compared by ANOVA followed by Tukey’s multiple comparison. Data are expressed as mean ± SEM

**Fig. 13:**
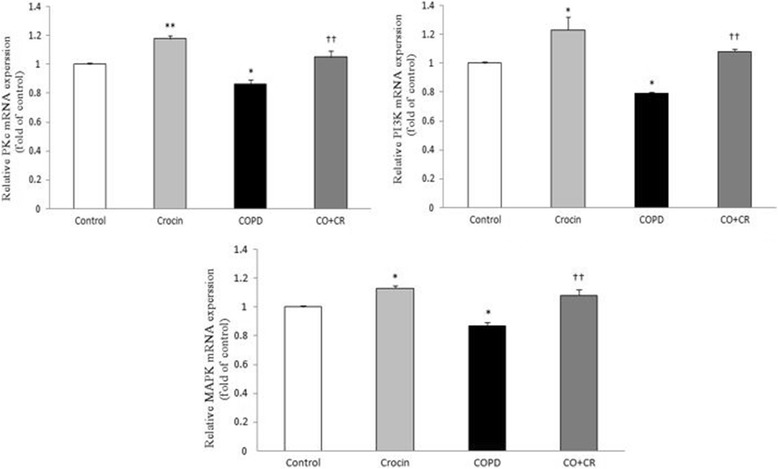
PKC, MAPK and PI3K mRNA expression in lung tissue of Control, Crocin, COPD and COPD plus crocin (CO + CR) rats. All the groups were statistically compared by ANOVA followed by Tukey’s multiple comparison. *P < 0.05 and **P < 0.01 vs. Control rats. ^††^*P* < 0.01 vs. COPD group. Data are expressed as mean ± SEM

According to Fig. 12, the gene expression of Nrf2 and GCLc significantly decreased after a 2-month cigarette smoke exposure compared with the corresponding controls. However, the mRNA expression of Nrf2 and GCLc significantly increased in crocin co-treatment CS rats compared with CS groups. The next step of the study was to confirm the effects of crocin on the expression of upstream Nrf2 regulator enzymes PKC, PI3K and MAPK. Accordingly, PKC, PI3K and MAPK mRNA levels in crocin plus CS group were significantly higher compared to these in the CS rats, suggesting that activation of Nrf2 pathway is achieved by increased expression of its regulator enzymes (Fig. 13).

**Fig. 14:**
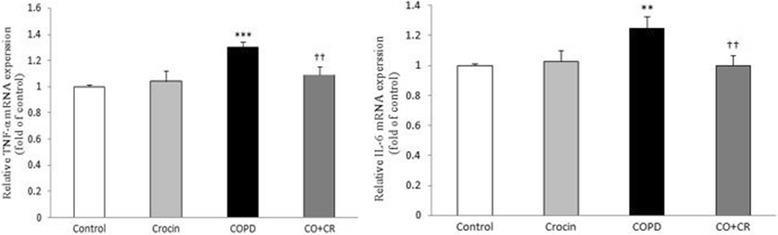
IL-6 and TNF-α mRNA expression in lung tissue of Control, Crocin, COPD and COPD plus Crocin (CO + CR) rats. All the groups were statistically compared by ANOVA followed by Tukey’s multiple comparison. **P < 0.01, ***P < 0.001 vs. Control group. ^††^*P* < 0.01 vs. COPD rats. Data are expressed as mean ± SEM

Also, to identify the mediators that may be involved in promoting the inflammatory response, we measured mRNA levels of IL-6 and TNF-α in lung tissue. The mRNA levels of IL-6 and TNF-α were significantly elevated at COPD group. In contrast, the cytokines gene expression in crocin plus CS group significantly decreased (Fig. 14).

### In vitro and in vivo cardiac parameters

To determine if CS-induced COPD has any in vivo effect on the heart, ECG under light anesthesia was performed to compare cardiac electrocardiogram parameters among all groups. Table 3 shows that the QTc interval and the QRS complex duration shortened in the CS rats. The heart rate increased in CS group, which was statistically significant. Table 2 shows that the RR interval, the QT interval and the ST segment shortened in the CS rats as compared to the control group, which was significant statistically.

**Table 2:** Arterial blood gas measurements in all groups

Groups	PO_2_(mmHg)	PCO_2_(mmHg)	pH
Control	105.50 ± 1.41	45.44 ± 2.42	7.340 ± 0.02
Crocin	100.87 ± 2.39	45.76 ± 1.98	7.362 ± 0.03
COPD	75.87 ± 3.08^***^	53.72 ± 3.65^**^	7.287 ± 0.08^**^
COPD + Crocin	98.62 ± 1.40^†††^	46.45 ± 1.90^†††^	7.330 ± 0.03^†††^

**P < 0.01 and ***P < 0.001, vs. control, ^†††^*P* < 0.001 vs. COPD. All the groups were statistically compared by ANOVA followed by Tukey’s multiple comparison. Data are expressed as the mean + SEM, n = 8

**Table 3:** Analyses of ECG waves among all groups

Parameters	Control	Crocin	COPD	COPD + Crocin
HR (bpm)	232.12 ± 11.46	237.50 ± 10.95	246.62 ± 15.81^*^	242.75 ± 6.13
RR Interval (S)	0.259 ± 0.01	0.253 ± 0.01	0.247 ± 0.006^*^	0.250 ± 0.01
QT Interval (ms)	61.15 ± 1.18	60.58 ± 0.76	58.65 ± 1.92^**^	60.82 ± 1.17^†^
QTc (ms)	119.69 ± 3.83	120.49 ± 1.99	118.94 ± 7.37	122.33 ± 2.87
QRS Complex (ms)	12.73 ± 0.45	12.51 ± 0.43	11.35 ± 0.84^***^	12.92 ± 0.44^††^
QRS Complex (mv)	0.53 ± 0.04	0.53 ± 0.06	0.49 ± 0.03	0.51 ± 0.04
ST Segment (ms)	14.39 ± 0.34	14.33 ± 0.45	13.71 ± 0.41^*^	14.52 ± 0.52^††^

*P < 0.05, **P < 0.01 and ***P < 0.001, vs. Control, ^†^*P* < 0.05 and ^††^*P* < 0.01 vs. COPD. All the groups were statistically compared by ANOVA followed by Tukey’s multiple comparison. Data are expressed as the mean + SEM, *n* = 8

**Table 4:** Hemodynamic parameters. All the groups were statistically compared by ANOVA followed by Tukey’s multiple comparison

Parameters	Control	Crocin	COPD	COPD + Crocin
LVDP (mmHg)	79.66 ± 7.31	83.61 ± 6.56	86.82 ± 3.74	82.37 ± 6.60
LVEDP (mmHg)	7.58 ± 0.46	7.63 ± 0.36	8.03 ± 0.25	7.75 ± 0.35
LVSP (mmHg)	87.25 ± 7.28	91.25 ± 6.67	95.37 ± 4.17	89 ± 5.78
dp/dt max (mmHg)	2864.25 ± 141.54	2934.25 ± 71.22	3078.87 ± 93.48^*^	2900.75 ± 144.72^†^
dp/dt min (mmHg)	− 2987.87 ± 147.05	− 3016 ± 118.03	− 3052.25 ± 84.74	− 3011.50 ± 92.42
RPP (mmHg/min)	22,195.12 ± 3120	24,103.75 ± 3201	27,518.75 ± 1227^**^	23,406.06 ± 3351^†^
Perfusion pressure (mmHg)	63.25 ± 5.11	64 ± 4.44	59.12 ± 6.46	61 ± 7.92
HR (bpm)	259 ± 16.93	263 ± 18.49	280 ± 21.61^*^	262 ± 21.76^†^

*P < 0.05 and **P < 0.01 vs. Control, ^†^*P* < 0.05 vs. COPD. Data are expressed as the mean + SEM, *n* = 8

To determine the intrinsic cardiac effects of CS-induced lung injuries, isolated hearts from all rat groups were then investigated for baseline cardiac hemodynamic parameters and contractile performance. Table 4 shows baseline functional parameters in isolated hearts in all groups. Assessment of the isolated hearts from CS rats showed significantly enhanced values for dP/dt_max_ and for the rate-pressure product. Co-treatment with crocin in CS group restored these cardiac responses towards values measured in control rats. Baseline perfusion pressure, left ventricular developed pressure and left ventricular end-diastolic pressure were not different between control, CS-exposed rats and crocin co-treated group.

### Right ventricular hypertrophy

**Fig. 15:**
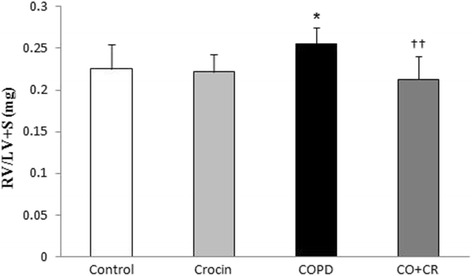
Fulton index in Control, Crocin, COPD and COPD plus crocin (CO + CR) rat. *P < 0.05 vs. Control and ^††^P < 0.01 vs. COPD. All the groups were statistically compared by ANOVA followed by Tukey’s multiple comparison. Data are expressed as the mean + SEM, *n* = 8

Cigarette smoke exposure was associated with right ventricular hypertrophy. RV/LV + S were significantly higher in the CS group than in the control rats (Fig. 15). Co-administration of crocin from the beginning of the CS exposure limited the development of right ventricular hypertrophy, as documented by a lower RV/LV + S in the co-treatment CS group than in the CS group.

## Discussion

While smoking-induced cardiovascular and pulmonary disorders are widely used in many experimental models, but any studies have been reported on models of COPD- induced cardiovascular dysfunctions. Indeed, there is no prior rat model that demonstrates a composite picture of COPD and cardiac dysfunction after chronic CS exposure. This is the first study to evaluation of cardiac electrocardiogram and function changes mediated by cigarette smoke induced- COPD in rat model.

Rodents are the most commonly used models in experimental studies. The rat is a favorable model, since measurable COPD changes which further progress can be detected after only 2 months of smoke exposure [38].

Pathological changes of COPD often occurred in the airway wall and lung parenchyma. In this experimental study, in cigarette smoke exposure rats, significant inflammation of the lung parenchyma was observed by chest x-ray, including diffuse patchy alveolar infiltration. In line with previous studies, it has been documented that cigarette smoke exposure for only two months was enough to induce alveolar wall destruction and airspace enlargement in rats [39]. Also, rats exposed to CS for 2 months exhibited significantly diffuse patchy alveolar infiltration, hyperinflation and disordered lung markings in chest x-ray, suggesting that CS-induced lung injuries mdel was successfully established. To further assess the pathological changes in the lung tissue, H&E staining was performed on all groups. While asthma affects the large and small airways, with COPD smaller airways and especially the alveoli are most involved. In fact, COPD is characterized by destruction of alveolar walls, leading to abnormally large air spaces within the lung. Therefore, Better recognition of small airway disease as a distinct pathological process holds the promise of a more site specific and targeted therapy [40]. Accordingly, in current study we assayed H&E staining of lung tissue and measured Mean linear intercept (MLI) as an index of alveolar airspace enlargement in response to CS and crocin. The results showed significant increases in MLI after 2 month cigarette smoke exposure. The lung tissue was significantly damaged in the rat model of CS induced- lung injuries, in which there were necrosis and degeneration of bronchial mucosal epithelium cells, serous fluid exudation from alveolar space, and inflammatory cell infiltration in bronchial wall and pulmonary consolidation. On the other hand, measurement of arterial blood gas exchanges were an excellent index of destruction in alveolar wall and gas exchange that confirmed establishment of lung injuries model in rats.

Cigarette smoke is a complex mixture of an estimated 5000 toxic chemical compounds; each puff of CS contains over 10^15^ free radicals, which include H_2_O_2_, reactive aldehydes, benzo pyrene and quinines [41]. CS plays a major etiological role in the pathogenesis of inflammatory airway disease, such as COPD.

Nuclear factor (erythroid-derived 2)-like 2 (Nrf2) is a regulator of cellular resistance to oxidative stress. The function of Nrf2 and its downstream target antioxidant genes have been shown to be important for cytoprotection against oxidant damage [42, 43]. Among the various antioxidant enzymes, the HO-1 is a major antioxidative and anti-inflammatory enzyme that is mainly regulated by Nrf2 activation [44]. Several studies demonstrated that up-regulation of this enzyme associated with its anti-inflammatory actions [45–47]. In this study, cigarette smoke down-regulated the mRNA expression of HO-1 in CS rats and co-treatment with crocin markedly induced HO-1 expression. These findings suggest that crocin may be a potential therapeutic candidate as an anti-inflammatory or an antioxidant agent. This finding was supported by modification of Nrf2 gene expression level.

Nrf2 has a pivotal role in the transcriptional regulation of phase II genes, including GCLc, thereby regulating GSH levels. GSH is an important lung antioxidant involved in maintenance of epithelial integrity, and its deficiency leads to airway injury and alveolar lung damage [48–51]. Previous studies have shown that Nrf2-deficient mice were more susceptible to CS-induced emphysema and oxidative stress [52], suggesting a protective role of Nrf2 in lung injury. In the light of this finding, we hypothesized that CS impairs Nrf2-mediated transcriptional regulation of GCLc and biosynthesis of GSH (ROS-Nrf2-GCLc-GSH pathway), whereas an agent that modulate Nrf2, attenuates CS induced oxidative stress and restores CS-depleted GSH via an Nrf2-GCLc pathway-dependent mechanism. In the current study, we tested our hypothesis by investigating the mechanism of CS-mediated downregulation of antioxidant genes and the protective role of crocin in rat lungs tissue. Since a variety of oxidants and free radicals are implicated in the pathogenesis of chronic obstructive pulmonary disease, it is possible that induction of antioxidant genes is an effective approach for protection against environmental factor-induced oxidative stress.

Crocin has been well known for its possible antioxidant and anti-inflammatory role and protective effects against oxidant damage [53]. In the present study, we also examined the ability of crocin to attenuate the CS-mediated oxidative stress and GSH depletion probably via an Nrf2- pathway, in cigarette smoke exposure rats. Our results demonstrate that 2-month CS exposure resulted in increased oxidative stress via increasing lipid proxidation (MDA), which was associated with decreased levels of Nrf2, GCLc gene expression, GSH amount and other antioxidant enzymes activity in the lung tissue. Recent studies have shown that acute cigarette smoke exposure increased [54] the levels of antioxidant enzymes in both rat lung and epithelial cell, but chronic exposure decreased these levels [55]. Researchers believe that with repeated exposures, Nrf2 becomes less sensitive to the smoke-derived oxidants, although no mechanism for this decreased sensitivity has been demonstrated [56]. On the other hand, Aldehydes formed by lipid peroxidation form protein carbonyl or present in CSE adduct with sulfhydryl groups of Nrf2/Keap1, thereby leading to modulation of these sulfhydryl groups. This could be the reason for failure to localize Nrf2 into the nucleus or retention of Nrf2 in the cytoplasm of epithelial cells at a later time point which leaded to decreased GCLc gene expression [9]. Our results are in agreement with earlier studies suggesting that CS induces oxidative stress by ROS generation in various organ tissues. The decrease in the levels of GSH could be due to the formation of GSH conjugates with electrophilic compounds present in CS and inhibitory action of CS on Nrf2 or its upstream regulator genes expression by interaction of electrophilic components of CSE with the cysteine group in the active site [57]^.^ Co-treatment with crocin, significantly decreased CSE-induced ROS production, which was associated with increased levels of GSH activity and GCLc expression, compared with treatment with CSE alone. It is possible that crocin attenuates CSE-mediated depletion of GSH levels by increasing the biosynthesis of GSH and also by scavenging CSE-induced ROS. Indeed**,** in this possible mechanism, Nrf2 is phosphorylated through upstream signaling kinases. When these pathways are activated, phosphorylated Nrf2 can be liberated from Keap1. In fact, crocin might activate upstream signaling kinases, such as protein kinase C, phosphatidylinositol 3-kinase, and mitogen-activated kinase to modify Nrf2 pathway. PKC, PI3K and MAPK as the strongest intracellular pro-survival signaling systems are the target of many antioxidant agents for providing survival signaling [58]. Thus, to elucidate the upstream signaling pathway involved in crocin-mediated Nrf2 activation and GCLc and HO-1 induction, we studied the effects of crocin on the expression of these kinases in cigarette smoke induced CS rats. We found that after 2-months cigarette smoke exposure, the expression of PKC, PI3K and MAPK suppressed and then activated in crocin plus CS rats. The suppressed of Nrf2 pathway appears to be a common property of cigarette smoke exposure in chronic conditions [10] as we demonstrated in CS rats. Our data specify that this suppressed in chronic oxidative stress may arise from the changing in expression of PKC, PI3K and MAPK. Post-translational modification affects Nrf2 differently by altering Nrf2’s interaction with Keap1 and Nrf2 localization. These alterations of Nrf2 protein are essential for this protein to transactivate its downstream genes [59]. PKC, PI3K and MAPK are responsible for posphorylation and regulation of Nrf2 pathway in several cells and organs including lungs. Previous studies showed that PKC and PI3K strongly activate the Nrf2 pathway but, the effects of MAPKs on Nrf2 signaling appear to depend on the specific MAPK [60]. In this study, we report the first evidence that Nrf2 is upregulated by PKC, PI3K and MAPK (ERK2) modifications in crocin co-treatment CS rats. In this context, some studies demonstrated that some kinases such as PKC and MAPK have been implicated in playing a role in pro-inflammatory pathways [61, 62]. In contrast, some other studies provided evidence to suggest that there is a differential PKC regulation of production of inflammatory factors. For example, Conventional PKCs regulate the pro-inflammatory cytokine in a MAPK-dependent manner whereas anti-inflammatory IL-10 is regulated by a PKCζ-dependent pathway. In addition, cross-regulation between these pathways exists [63–65]. Taken together, these observations suggest that anti-inflammatory and pro-inflammatory cytokines are differentially regulated by these kinases isoform.

In summary, in this part we have specified the molecular mechanisms of regulation of Nrf2 pathway by crocin during the chronic cigarette smoke exposure induced lung injuries. The mechanism of changing in these kinases is still unclear. However, it has been reported that the activity of this kinases can be affected by several factors including alteration of calcium homeostasis [66].

Many of the intracellular signaling pathways triggering and/or driving the release of these inflammatory mediators is sensitive to oxidative stress. Elevated levels of ROS have been further implicated in initiating various inflammatory processes in the lungs through the activation of transcription factors, signal transduction and gene expression of pro-inflammatory mediators which correlates with pathological changes in airway wall and lung parenchyma [67]. In this study, the alteration of the inflammatory cells and total protein in BALF and also, gene expression and levels of TNF-α and IL-6, as important pro-inflammatory cytokines involved in immunoregulation and inflammation in lung tissue and BALF, was investigated for an in-depth evaluation of the inflammatory changes of lung in cigarette smoke induced COPD rat model. These findings suggest that total protein and inflammatory cytokines plays a pivotal role in cigarette smoke induced lung injury. In accordance with previous studies, exposure to cigarette smoke caused elevated in TNF-α and IL-6 gene expression in lung tissue and enhanced levels of these cytokines in the BALF of rats compared to the control group which was correlated with the increase in inflammatory cells in bronchoalveolar lavage fluid, suggesting that increased inflammatory factors production increases COPD severity. However, the co-administration of crocin to the CS rats decreased cytokines gene expression and levels (IL-6 and TNF-α) and the number of inflammatory cells (macrophages and lymphocyte cells), ameliorated lung tissue injury. These effects may be related to the inhibition of releasing IL-6 and TNF-α by crocin, resulting in a decrease in the inflammatory cells recruitment responsible for lung injury in cigarette smoke exposure rats.

Chronic obstructive pulmonary disease is a chronic inflammatory condition of the lungs whose manifestations are linked to other systemic diseases, among which an increased risk of cardiovascular disorder is that, contributes significantly to both morbidity and mortality in COPD [68]. Smoking is the causative factor in the majority of individuals with COPD and in the development of coronary artery disease [69]. It is, therefore, difficult to show in a COPD population that the increased risk of cardiovascular disease is due to COPD alone, because COPD and smoking are inextricably linked [70]. However, to determine if CS-induced lung injuries established model has any in vivo and in vitro effect on the heart, ECG recording and isolated-perfused rat heart were performed to compare cardiac electrocardiogram and function between COPD and control rats.

To our knowledge, this is the first study that demonstrates significant deleterious effects of CS- induced COPD on the general cardiac hemodynamic, function and electrocardiogram in a rat model. We observed that CS-induced lung injuries resulted in significant shortening of the QRS complex, whereas the other waves did not show much change. Our results showed shortening of the QTc interval in CS rats, which was not statistically significant. These results were in accordance with the Karjalainen et al.’s study [71] which explained that a shortened QT interval and ST segment were as a risk factor for smokers and could cause death. The shortened ST segments and QT interval warn that there may be shortening in the ventricular filling phase, during which the coronary supply occurs. This may lead to an insufficient myocardial perfusion, which may lead to ischemic episodes. Such altered ventricular electrical activities, like a shortened ST segment and QRS complex also predisposes COPD patients to episodes of arrhythmia. The increased heart rate which was shown in our study probably was due to an increased catecholamine secretion from the adrenal medulla [72]. There are studies whose results demonstrate that cigarette smoke also may significantly increase levels of epinephrine and norepinephrine [73]; however, we did not assess plasma and urinary catecholamines in rats. All the above changes in our study were the result of the 2 months cigarette smoke exposure, which could lead to cardiovascular disorders easily identified by the wave duration in electrocardiography. COPD can increase the risk of sudden cardiac deaths. In the long term, the mortality which is caused by COPD is due to either coronary artery disease or electrophysiological disturbances, which lead to arrhythmias.

COPD also can impair cardiac hemodynamic and function. We observed an adverse effect of cigarette smoke exposure on the in vitro baseline cardiac contractile function at the organ level. It is important to note that hypoxemia can influence cardiac function [74]. We performed ABG analysis as mainly used to evaluate and monitor gas exchange in the lungs. Two months of CS exposure showed a significant decrease in PO_2_ in CS rats as opposed to the control group. Hypoxia is a potent stimulus for the generation of ROS and also induces hemodynamic stress [70]. Under physiological conditions, ROS are important mediators of a wide variety of cell functions. However, excessive generation of ROS and the fall in antioxidant defenses can lead to cellular oxidative stress. In our study, isolated hearts from CS rats showed significantly enhanced values for dP/dt_max_ and for the rate-pressure product. While co-treatment with crocin in CS-induced lung injuries rats restored these cardiac responses towards values measured in CS group. Several studies have also shown that hypoxia can lead to cardiac dysfunction in rats. Some adverse effects include altered metabolism and myocardial structure, a change in cardiac performance and heightened cardiac susceptibility to adult ischemic injury [75]. These experimental studies are of substantial clinical relevance, as it has now also been reported that, hypoxia modulated by CS induced-COPD show changes in cardiac morphology (RV hypertrophy) and function [52]. Here, we show that COPD has permanently altered the mechanical properties of the myocardium. An increase in the rate-pressure product is associated with increased myocardial work load and oxygen consumption and an increase in dP/dt_max_ is an established index of increased myocardial contractility [76]. It is possible that the increase in myocardial contractility occurs in response to the increased afterload derived from the increased peripheral vascular impedance. Sustained increases in myocardial contractility can strongly be associated with cardiovascular disease, and this cardiac phenotype is a known predictor of eventual heart failure in humans [76].

Our results also suggested that the effects of CS-induced lung injuries on the heart of CS rats can be prevented by crocin co-treatment during CS exposure, providing new evidence for the mechanism driving the developmental process of heart dysfunction by COPD to be also secondary to oxidative stress. Co-treatment with crocin may prevent hypoxia influences on the heart triggered by COPD-associated lung dysfunction by ameliorating oxidative stress via Nrf2 pathway. Further studies are needed to investigate the therapeutic effects of crocin. In this regard, using novel approaches such as Q3D-compartment multiscale model can be used to modeling the drug transport across the layers and along the lung and optimizing the target-specific drug delivery and increasing the localized bio-availability [77, 78].

**Fig. 16:**
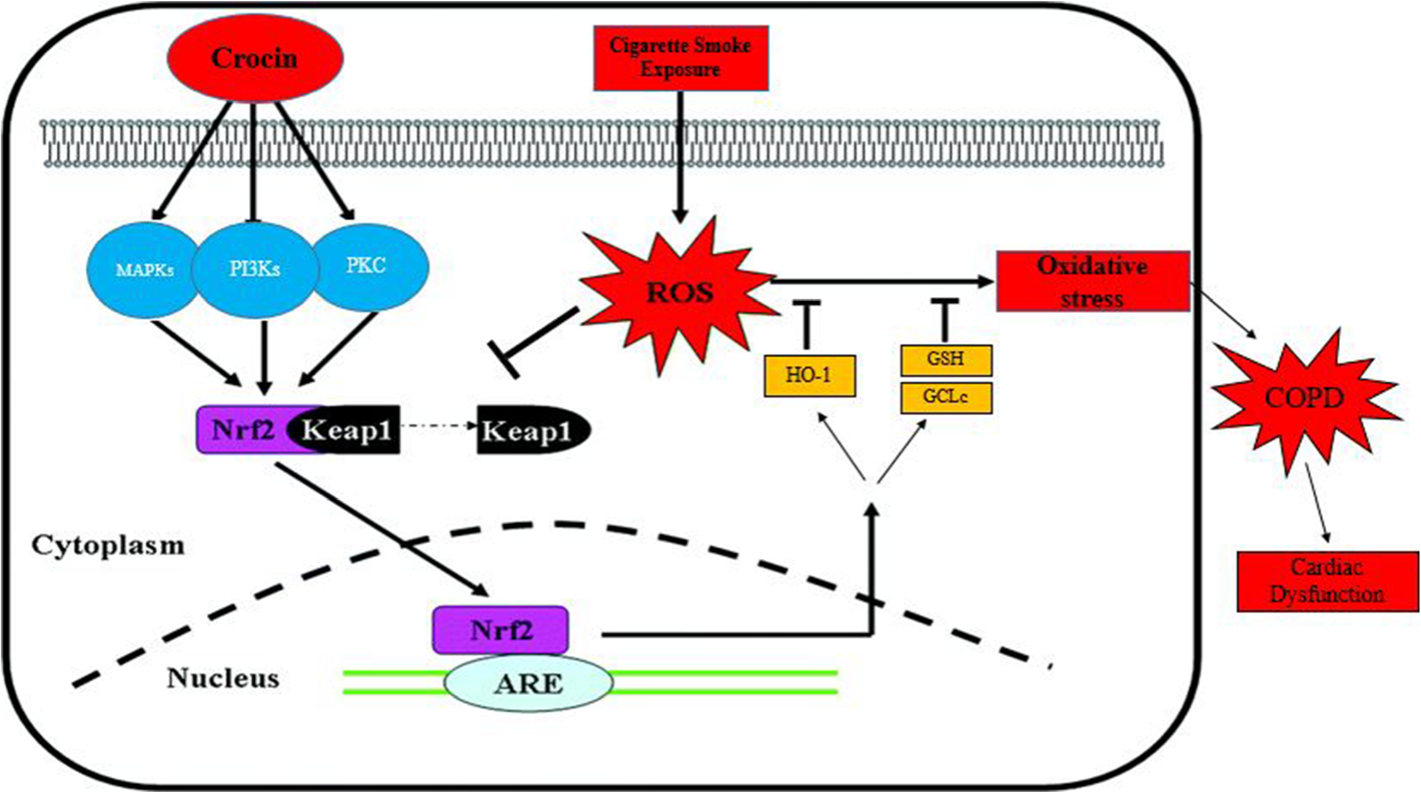
Schematic representation depicting the mechanism of cigarette smoke induced- COPD via Nrf2 pathway and protective role of crocin

In summary, our study was designed to investigate the effects of a 2-months cigarette smoke exposure in establishment of rat lung injuries model and provide substantial data regarding the pathogenic oxidative stress processes in this model. We sought to demonstrate whether the CS exposure-dependent alterations in lung morphology and parameters and cardiac disorder in COPD are concurrent with oxidative stress, particularly, in Nrf2 pathway (both upstream and downstream genes) (Fig. 16). Although our findings provide convincing insights into a versatile rat model of chronic smoking, the exposure time was moderate, and by designing only 2 months CS exposure was tested. Further studies with different levels of CS exposure and longer periods would be useful in further determining the relationship between duration and dose of exposure in the process of chronic cigarette smoking induced COPD flowed by cardiac disorders. Along with the future studies, this rat model of smoking-induced COPD should be useful to determine the mechanisms of COPD-induced disease and approaches for early detection and prevention.

## Conclusion

The present in vivo rat model provides convincing evidence that chronic CS exposure induces significant alterations in pulmonary system that mediated cardiac function, structure, and electrocardiogram with cellular oxidative stress and decreased PKC, PI3K, MAPK, Nrf2 and GCLc genes expression decreasing antioxidant enzymes level. On the other hand, our results provided the evidence that crocin co-administration is protective against lung injury caused by CS cigarette smoke exposure and related cardiac dysfunction. Our data provided the evidence that these protective effects are probably related to Nrf2 pathway (both upstream and downstream genes). Further molecular and cellular studies in this model should enable the determination of important mechanistic insights in CS-induced diseases.

## Abbreviations

ABG: Arterial blood gas
BALF: Bronchoalveolar lavage fluid
CAD: Coronary artery disease
COPD: Chronic obstructive pulmonary disease
CS: Cigarette smoke
CSE: Cigarette smoke exposure
GCLc: Glutamate-cysteine ligase catalytic subunit
GPX: Glutathione Peroxidase
GSH: Glutathione
MDA: Malondialdehyde
Nrf2: Nuclear erythroid-related factor 2
MAPK: Mitogen-activated protein kinases
PKC: Protein kinase C
PI3K: Phosphoinositide-3-kinase
PBS: Phosphate-buffered saline
ROS: Reactive oxygen species
TNF-α: Tumor necrosis factor
